# *gbpA* as a Novel qPCR Target for the Species-Specific Detection of *Vibrio cholerae* O1, O139, Non-O1/Non-O139 in Environmental, Stool, and Historical Continuous Plankton Recorder Samples

**DOI:** 10.1371/journal.pone.0123983

**Published:** 2015-04-27

**Authors:** Luigi Vezzulli, Monica Stauder, Chiara Grande, Elisabetta Pezzati, Hans M. Verheye, Nicholas J. P. Owens, Carla Pruzzo

**Affiliations:** 1 Department of Earth, Environmental and Life Sciences (DISTAV), University of Genoa, Genoa, Italy; 2 Oceans and Coastal Research, Department of Environmental Affairs (DEA) and Marine Research Institute, University of Cape Town (UCT), Cape Town, South Africa; 3 Sir Alister Hardy Foundation for Ocean Science (SAHFOS), Plymouth, United Kingdom; University of Padova, Medical School, ITALY

## Abstract

The *Vibrio cholerae* N-acetyl glucosamine-binding protein A (GbpA) is a chitin-binding protein involved in *V*. *cholerae* attachment to environmental chitin surfaces and human intestinal cells. We previously investigated the distribution and genetic variations of *gbpA* in a large collection of *V*. *cholerae* strains and found that the gene is consistently present and highly conserved in this species. Primers and probe were designed from the *gbpA* sequence of *V*. *cholerae* and a new Taq-based qPCR protocol was developed for diagnostic detection and quantification of the bacterium in environmental and stool samples. In addition, the positions of primers targeting the *gbpA* gene region were selected to obtain a short amplified fragment of 206 bp and the protocol was optimized for the analysis of formalin-fixed samples, such as historical Continuous Plankton Recorder (CPR) samples. Overall, the method is sensitive (50 gene copies), highly specific for *V*. *cholerae* and failed to amplify strains of the closely-related species *Vibrio mimicus*. The sensitivity of the assay applied to environmental and stool samples spiked with *V*. *cholerae* ATCC 39315 was comparable to that of pure cultures and was of 10^*2*^ genomic units/l for drinking and seawater samples, 10^1^ genomic units/g for sediment and 10^2^ genomic units/g for bivalve and stool samples. The method also performs well when tested on artificially formalin-fixed and degraded genomic samples and was able to amplify *V*. *cholerae* DNA in historical CPR samples, the earliest of which date back to August 1966. The detection of *V*. *cholerae* in CPR samples collected in cholera endemic areas such as the Benguela Current Large Marine Ecosystem (BCLME) is of particular significance and represents a proof of concept for the possible use of the CPR technology and the developed qPCR assay in cholera studies.

## Introduction

*Vibrio cholerae* is the causative agent of cholera, an enteric disease which affects the intestinal tract, characterized by severe diarrhea, vomiting and dehydration. Cholera continues to be a major cause of morbidity and mortality around the world as every year 3–5 million people are infected with cholera and 100,000–120,000 people die from the disease [1].

*V*. *cholerae* is an environmental bacterium which thrives in brackish and estuarine water around the world mostly in association with a variety of environmental reservoirs and/or hosts such as plankton, bivalves, other aquatic animals and plants, and aquatic sediments [2]. The role of these substrates in cholera endemicity and/or the transmission of the disease to humans is well documented [2,3] and monitoring the bacterium in environmental sources is of utmost importance to understand its ecology and spread, and to take preventive measures for its control [4].

Culture-dependent methods used to detect and classify *V*. *cholerae* are laborious and time-consuming [5] as they require prolonged incubation and growth on selective media to reduce the number of non-target organisms [6, 7]. Alternative, more rapid, specific and sensitive molecular-based techniques have been developed recently. The added value of molecular procedures is the capability to detect *V*. *cholerae* in DNA extracted from environmental samples, also when present in the viable but not culturable (VBNC) form, a dormant state that allows bacteria to survive and persist in the natural environment under unfavorable conditions [8,9].

Among the techniques reported in the literature some procedures have been developed for *V*. *cholerae*/*V*. *mimicus* clade detection [10, 11], with *V*. *mimicus* phylogenetically being the most closely related species to *V*. *cholerae*, while other methods are multiplex polymerase chain reaction assays for simultaneous detection of *V*. *cholerae* and other pathogens [12,13]. However, most of these analysis procedures are non-quantitative [14] or were only tested on few types of samples (mainly pure culture, stool or water samples) [15–20].

Generally, direct PCR-based analyses of complex environmental samples are problematic due to the low level of *V*. *cholerae* in environmental matrices, the concomitant large number of non-target microorganisms and the presence of PCR inhibitors [21]. As a consequence, there is an obvious lack of a rapid, sensitive, specific and quantitative method to detect *V*. *cholerae* species in these samples. This particularly holds true for detection of the bacterium in highly problematic samples such as formalin-fixed samples where DNA can be damaged (e.g. fragmented) and the PCR reaction can be hampered by the presence of inhibitors [22]. These types of samples such as natural history collections and/or other repository collections from public and private institutions worldwide are of outstanding value to a wide range of studies, including genetic, evolutionary, biogeographic, ecological and epidemiological studies, especially with the recent development of molecular biology techniques [23,24]. For instance, insights into the ecology of vibrios in coastal marine environments have recently been obtained by the retrospective analysis of formalin-fixed plankton samples collected in the last 60 years by the Continuous Plankton Recorder (CPR) Survey [25].

To provide a method for the detection and quantification of *V*. *cholerae* in problematic samples we identified a new taxonomic marker, namely the *gbpA* gene encoding for the N-Acetylglucosamine (GlcNAc) binding protein A of *V*. *cholerae*. GbpA is a 53 kDa extracellular secreted and surface-associated protein that has recently been shown to be involved in *V*. *cholerae* attachment to environmental chitin surfaces and cultured intestinal epithelial cells via the same GlcNAc binding specificity [26–28]. In a previous study we evaluated the distribution of the gene in 488 *V*. *cholerae* strains of environmental and clinical origin, belonging to different serogroups and biotypes and found that *gbpA* is always present in *V*. *cholerae* [28]. In addition, *in silico* analysis performed on full sequences of the gene in different *Vibrio* species revealed that *gbpA* differs substantially within the *Vibrio* genus whilst it is highly conserved within the species *V*. *cholerae* [28].

As a follow up to our previous observations, this study developed a new, rapid, sensitive and quantitative species-specific Taq-Man based Real-Time PCR protocol targeting the *gbpA* gene of *V*. *cholerae*. The protocol is optimized for capillary PCR and works efficiently for the detection of the bacterium in problematic environmental samples. In addition, being based on the amplification of a 206 bp small DNA fragment, the developed protocol is suitable for the detection of *V*. *cholerae* in highly damaged samples such as historical formalin-fixed CPR samples.

## Materials and Methods

### Ethics Statement

For the collection of freshwater and marine sediment samples local competent authorities (Municipality of Goro and Municipality of Genoa) were informed and allowed the sampling for research purpose only. For the collection and use of the bivalve *Mytilus galloprovincialis* no specific permits were required according to Italian legislative decree 4 March 2014, n. 26 and EU directive 2010/63/UE (Legislation for the protection of animals used for scientific purposes). In any case, we declare that the present study did not involve endangered or protected species and that stress was minimized during both animal samplings and experiments. Mussels were purchased from an aquaculture farm (Arborea, Italy) and sacrificed in the laboratory by laboratory staff for the purpose of the study. To ameliorate the suffering of the animals the method of sacrifice consisted in the cutting of the posterior adductor muscle followed by rapid freezing of tissues (this practice is known to ameliorate the suffering of the bivalve without the use of anesthetics/analgesics which are not recommended for this type of study).

For the collection and use of fecal samples written informed consent was obtained from the parents of a healthy 2-years old male donor according to the Italian law. All work with fecal human samples was approved by the competent ethics committee (Sezione 1, Comitato Etico Regione Liguria) on 09/12/2014, approval number 501REG2014.

### Bacterial strains, culture conditions and DNA extraction

A total of 129 bacterial strains belonging to different *Vibrio* and other related species were used in this study (S1 Table). Strain *V*. *cholerae* O1 El Tor N16961 (ATCC 39315) was used as the reference strain. Bacterial cells were grown in Luria Bertani (LB: yeast 5 g/l, bacto-tryptone 10 g/l, NaCl 10 g/l) broth or agar, and on Thiosulfate-citrate-bile salts-sucrose TCBS (Lab M, United Kingdom) agar at 37°C. After overnight growth, cells were harvested by centrifugation (4500 g for 10 min), washed twice with phosphate-buffered saline (PBS: NaCl 8 g/l; KCl 0.2 g/l; Na_2_HPO_4_.12H_2_O 3.62 g/l; KHPO_4_ 0.24 g/l) or artificial sea water (ASW: NaF 1.9 mg/l; SrCl_2_.6H_2_O 13 mg/l; H_3_BO_3_ 20 mg/l; KBr 67 mg/l; KCl 466 mg/l; CaCl_2_.2H_2_O 733 mg/l; Na_2_SO_4_ 2.66 g/l; MgCl_2_.6H_2_O 3.33 g/l; NaHCO3 133 mg/l; NaCl 27.65 g/l; pH 8) and resuspended in the appropriate buffer, according to the assay to be performed, to reach a final concentration of 0.2 OD_600_. For each strain, DNA was extracted from pure culture by using the High Pure PCR Template Preparation Kit (Roche Diagnostics, Milan, Italy) according to the manufacturer’s instructions.

### PCR primers and probe development

The primers pairs: gbpA-seq1F (5-tca ctc tga act gcg tct gg-3) and gbpA-seq1R (5-ttg gtt agc gtc tca gag tca a-3) and gbpA-seq2F (5-cac tcg cgt gtt tga taa cg-3) and gbpA-seq2R (5-gtg gag agg tag cca ctg ga-3) amplifying positions 1–767 and 690–1458 of the *gbpA* gene of *Vibrio cholerae* N16961 (GenBank accession number: EU072441.1) respectively, were previously designed to amplify the entire *gbpA* open reading frame (ORF) in 36 representative *V*. *cholerae* strains [28]. Amplified fragments from the PCR reaction were purified with the High Pure PCR Template Preparation Kit (Roche Diagnostics, Milan, Italy) and sequenced by using the Sanger dye-terminator method [28]. The *gbpA* gene sequences were then aligned using the BIOEDIT software [29] and primers and a probe were designed to target a species-specific region of the gene showing the lowest sequence variability (>99%) within *V*. *cholerae* using Primer3 [30, 31]. The chosen primer and probe sequences for this qPCR assay were Vc gbpA F (5-ccg cag ctt cct tct aca ac-3), V.c gbpA R (5-ggc ttt ggt tag cgt ctc ag-3) and V.c gbpA pr (5-FAM-aac cca gca ggt caa atc att cca agt a-BBQ) probe. The Nucleotide Basic Local Alignment Search Tool (BLASTn) and Primer-BLAST tool [32] were used to check that the Vc gbpA F, V.c gbpA R and and V.c gbpA pr did not match any non-target *Vibrio* species. The primers and probes were synthesized by Tib Molbiol srl (Genoa, Italy).

### Construction of standard curves for qPCR and Interference assays

To determine the limits of detection, linear ranges, and amplification efficiencies of the qPCR assay, a standard curve, based on accurately quantified genomic DNA of *V*. *cholerae* ATCC 39315 strain (Genetic PCR solution TM, Alicante, Spain), was constructed. The genomic standard (GS) was purified and checked for the absence of PCR inhibitors by constructing a standard curve plot (inhibition plot). The concentration was determined by fluorometry and expressed as genomic units/μl (GU/μl). GS was serially diluted to prepare solutions containing 10^6^ to 10^1^ genome copies per reaction (GU/rx). For each qPCR assay, a total of three separate runs were performed (each run in triplicate) to determine intra-assay and inter-assay reproducibility (assessed by computing the coefficient of variation, %CV). The limit of detection of the qPCR assay was determined as the lowest concentration at which 100% of the replicates were detected. To assess interference from non-target DNA, increasing concentrations of purified genomic DNA of *V*. *cholerae* ATCC 39315 (from 10^1^ to 10^4^ GU/μl) were also quantified by the qPCR in the presence of purified DNA from *V*. *mimicus* UM 6812 that was added to the qPCR reaction mixture at a concentration of 0.5 ng/rx (corresponding to 8x10^4^ GU/rx).

### qPCR amplification

The qPCR amplification protocol was set up on a Light Cycler 1.5 instrument (Roche Diagnostics, Milan, Italy) using a Light Cycler TaqMan Master Mix chemistry [33]. Amplification reaction mixtures (20 μl) contained: 1x TaqMan Master Mix; 200 nM primers; 25 nM probe; DNA sample (0.2–2 ng/μl) and five microlitres of DNA template. The PCR program used was as follows: initial denaturation at 95°C for 10 min, subsequent 45 cycles of denaturation at 95°C for 10 s, annealing at 59°C for 20 s and elongation at 72°C for 1 s, followed by a cooling step at 40°C for 30 s. Amplicons were visualized by agarose gel electrophoresis using ethidium bromide solution. Accurately quantified genomic DNA of *V*. *cholerae* ATCC 39315 strain was used as a standard (Genetic PCR solution, Alicante, Spain). For quantification, the log of the number of genome units (GU) of a dilution series of the sample was plotted versus the cycle number at which the fluorescent signal increased above threshold (Cq-value).

### qPCR studies with artificially spiked environmental and stool samples

#### Drinking and seawater samples

One litre of tap water collected at the laboratory of microbiology of the University of Genoa and sea surface water (ASW) obtained by reconstituting Sea Salts (Sigma—Aldrich) with demineralized hypo-osmolar water to 30‰ final concentration was spiked with different concentrations of *V*. *cholerae* ATCC 39315 (from 10^6^ to 10^3^ cells per litre) and subsequently filtered onto 0.22 μm-pore-size Millipore membrane (47 mm in diameter) (Millipore, Milan, Italy). DNA from filter-bound material was extracted by using Rapid Water DNA Isolation Kit (MoBio Laboratories, Solana Beach, CA, USA) according to the manufacturer’s instructions.

#### Aquatic sediment samples

Aliquots of 1 g of freshwater (Brugneto Lake, Genoa, Italy) and marine sediment (Sacca di Goro, Ferrara, Italy) were spiked with different concentrations of *V*. *cholerae* ATCC 39315 (from 10^6^ to 10^1^ cells per gram). The absence of *V*. *cholerae* in these samples was previously evaluated and confirmed by qPCR. A total of 0.5 g from each sample was used for DNA extraction by using Ultra Clean soil DNA kit (MoBio Laboratories, Solana Beach, CA, USA) according to manufacturer’s instructions.

#### Bivalve samples

Freshly harvested mussels (*Mytilus galloprovincialis*) obtained from a fishfarm in Sardinia (Mediterranean Sea, Italy) were transported on ice to the Laboratory of Microbiology of the University of Genoa, stored at refrigeration temperature (5°C), and subjected to microbiological analysis within 4 to 8 h. The absence of *V*. *cholerae* in bivalve samples was previously evaluated and confirmed by qPCR. Aliquots of 1 g of *V*. *cholerae*-free mussel flesh homogenate were then spiked with different concentrations of *V*. *cholerae* ATCC 39315 (from 10^6^ to 10^1^ cells per gram). A total of 0.25 g from each sample was used for DNA extraction by using High Pure PCR Template Preparation Kit (Roche Diagnostics, Milan, Italy) according to manufacturer’s instructions.

#### Stool samples

A single fecal sample (1 g) was obtained from a healthy 2-years old male donor. The sample was spiked with different concentrations of *V*. *cholerae* ATCC 39315 (from 10^6^ to 10^1^ cells per gram). A total of 0.5 g from each sample was used for DNA extraction by using Ultra Clean soil DNA kit (MoBio Laboratories, Solana Beach, CA, USA) according to manufacturer’s instructions.

For the construction of standard curves, each DNA sample from the different above mentioned preparations was run in triplicate by the qPCR method.

### qPCR studies with artificial formalin-fixed samples

*V*. *cholerae* O1 El Tor (ATCC 39315) was grown in Luria Bertani broth as previously described and resuspended in PBS to reach a final concentration of 10^8^ CFU/ml. 10ml of the suspension was then spiked with 400 μl of neutral buffered Formaldehyde (4% final concentration) and serially diluted to prepare solutions containing 10^6^ to 10^1^ GU/rx. Genomic DNA was extracted after 1, 2, 4 and 8 weeks using the protocol reported in Vezzulli *et al*. [25] for the analysis of CPR samples (see below). For the construction of standard curves, each DNA sample was run in triplicate by the qPCR method.

### qPCR studies with artificially fragmented DNA samples

Genomic DNA was extracted from pure culture of *V*. *cholerae* O1 El Tor (ATCC 39315) as previously described. 1.2 μg of genomic DNA was artificially fragmented using a Proven Covaris AFA shearing process (Covaris Ltd, Brighton UK) generating an homogenous pool of fragments having an average size of 276 bp. Fragmented genomic DNA samples were purified using an Agencourt AMPure XP system (Beckman Coulter s.r.l. Milan, Italy) following the manufacturer’s instructions and run on an Agilent Bioanalyzer 2100 (Agilent, Palo Alto, CA, USA) using the High Sensitivity DNA kit (Agilent Technologies). Each DNA sample was then run in triplicate by the qPCR method.

For comparison, the same samples were also analyzed by the qPCR protocol described by Chun *et al*. [15] targeting the 16S-23S rRNA Intergenic Spacer Regions (ISR) for the detection and quantification of *V*. *cholerae*. The Promega GoTaq qPCR Master Mix kit, optimized for use with glass capillaries and containing a hot start polymerase, was used as the master mix base for all reactions. Primers used were: prVC-F 5-tta agc gtt ttc gct gag aat g- 3 and prVC-R 5 agt cac tta acc ata caa ccc g-3 [15]. Each reaction mixture contained 0.2 umol of each primer in a final volume of 20 μl. The PCR programme used was as follows: initial denaturation at 95°C for 2 min, subsequent 45 cycles of denaturation at 95°C for 15 s, annealing at 58°C for 30 s and elongation at 72°C for 45 s, followed by final elongation at 72°C for 30 s. PCR runs were analyzed directly in the LightCycler using melting analysis and the analysis software provided with the instrument.

### qPCR studies with historical formalin-fixed samples (CPR samples)

Formalin-fixed plankton samples collected in different coastal areas in the last 60 years by the Continuous Plankton Recorder (CPR) Survey were retrieved from the CPR archive in Plymouth (England). The CPR is a high-speed plankton sampler designed to be towed from commercially operated ships of opportunity over long distances [34]. Sampling takes place in the surface layer (7 m) and plankton is collected on a band of silk (mesh size 270 μm) that moves across the sampling aperture at a rate proportional to the speed of the towing ship. On return to the laboratory, the silk is removed from the device and divided into individual samples that are stored in airtight plastic boxes in buffered formalin (usually comprising 4–10% buffered formalin) [36].

To test the performance of the qPCR method to detect *V*. *cholerae* O1, O139, Non-O1, and Non-O139 in historical formalin-fixed samples three different sets of CPR samples were used. The first set included two samples: samples 413R and 485R collected in the North Sea off the river Rhine estuary in 1998 and 2004, respectively (S2 Table). Both of these samples were previously analysed by 16SrDNA amplicon pyrosequencing, revealing the presence of read sequences showing >95% identity to *V*. *cholerae* [25]. A second set included 5 samples collected in the early years of the CPR Survey in different coastal areas of the North Sea and North Atlantic Ocean: 2EB collected off Nova Scotia in 1961; 11IN2 collected in the Irish Sea in 1966; 228A collected near the Shetland Islands in 1966; 157SB collected in the Bay of Biscay in 1971 and 157SB collected off the Iberian Coast in 1971 (S2 Table). Finally, a third set included 18 samples collected by the South African CPR sister survey in the Benguela Current Large Marine Ecosystem (BCLME) region during the inaugural BC-CPR survey (Luanda, Angola—Durban, South Africa) in September 2011: 4CT 2, 4CT 4, 4CT 6, 4CT 24, 4CT 26, 4CT 28, 4CT 38, 4CT 40, 4CTend, 6CT 18, 6CT 20, 7CT 20, 7CT 22, 7CTend, 8CT 26, 8CT 28, 8CT 30, 8CT 32 (S2 Table).

Genomic DNA was extracted from CPR samples using the protocol previously described in Vezzulli *et al*. [25]. Briefly, for each sample, the filtering silk was cut into five replicate (1 cm^2^) sections. Each section was placed in a sterile tube, after which 25 ml of TE buffer (10 mM Tris-HCl, 1mM EDTA, pH 8.0) were added and vortexed to detach plankton from the silk mesh. Samples were left to rest at room temperature for 24 h; the plankton suspension was then gently centrifuged (400g) and the pellet transferred to a sterile microcentrifuge tube. Fifty μl of lysozyme (2 mg/ml in 10 mM Tris-HCl, pH 8.0) was added to the sample that was then vortexed vigorously for 1 min. After the addition of 180 μl 10% sodium dodecyl sulphate and 25 μl proteinase K (10 mg/ml), the sample was vortexed for 30 sec. The sample was then incubated at 56°C for 1 h, heated at 90°C for 1 h in a dry-block heater, vortexed for 10 sec and centrifuged at 12000 g for 3 min. After addition of 200 μl guanidine hydro-chloride lysis solution and 200 μl ethanol, the sample was centrifuged (12000 g for 10 sec). The supernatant was then transferred to QIAamp MinElute column (Qiagen, Valencia, CA, USA) and processed according to the manufacturer’s recommendation. The retained DNA was purified with QIAquick PCR purification columns (Qiagen spa, Milano, Italy) up to a final yield of 1–7 mg/ml. The LightMix Modular PhHV spiked Extraction Control (Roche Diagnostics, Milan, Italy) was used to test for the presence of DNA inhibitors in representative CPR samples (see below). Each DNA sample was run in triplicate by the qPCR method. A “Vibrio relative abundance index” (VAI), defined as the ratio of *Vibrio* spp. cells to the total number of bacterial cells assessed by real-time PCR using genus-specific (Vib1 5-ggc gta aag cgc atg cag gt-3 and Vib2 5-gaa att cta ccc ccc tct aca g-3) [35] and bacterial universal primers (967f 5-caa cgc gaa gaa cct tac c-3 and 1046r 5-cga cag cca tgc anc acc-3 [36] respectively, was also measured on the 18 samples collected in southern Africa in 2011 following the protocol previously described in Vezzulli *et al*. [25].

## Results and Discussion

### Quality evaluation of *V*. *cholerae* primers and Taq-Man probe

Species-specific PCR primers (VcgbpA F; VcgbpA R) and probe (VcgbpA pr) for the detection and quantification of *V*. *cholerae* were designed within the region of the *gbpA* gene showing the lowest sequence variability in *V*. *cholerae* species according to bioinformatics analysis. The primers target a small DNA fragment (206 bp) amplifying positions 566–771 of the *gbpA* gene of *V*. *cholerae* O1 El Tor (ATCC 39315). The quality of the developed primers and probe was evaluated in terms of *in-silico* target species coverage (sensitivity) and specificity against the GenBank NIH genetic sequence database [37], using BLASTn and the Primer-BLAST tool [32]. The primers and probe cover almost 100% of *V*. *cholerae* target sequences within the reference database and showed a good performance in term of specificity as BLAST results did not show any significant homology to other published sequences in the GenBank, DDBJ, and EMBL databases. Interestingly, *V*. *mimicus*, a highly related species to *V*. *cholerae*, was not completely matched by the primers and probe.

### Evaluation of *V*. *cholerae* qPCR specificity with pure cultures

A first set of trial qPCR experiments was conducted on genomic DNA extracted from pure culture of *V*. *cholerae* ATCC 39315 for setting optimal annealing temperatures and primer concentrations (corresponding to the highest qPCR specificity without reduction in yield) that were determined to be 59°C and 200 nM, respectively. qPCR specificity was evaluated by testing the developed primers and protocol on genomic DNA purified from 79 *V*. *cholerae* strains of environmental and clinical origin, belonging to different serogroups and biotypes, and a total of 48 strains belonging to other *Vibrio* species (4 *V*. *aestuarianus*, 4 *V*. *alginolyticus*, 1 *V*. *anguillarium*, 2 *V*. *corallilyticus*, 3 *V*. *harveyi*, *1 V*. *metecus*, 2 *V*. *mimicus*, 20 *V*. *parahaemolyticus*, 1 *V*. *parilis*, 5 *V*. *splendidus*, 2 *V*. *tapetis*, 2 *V*. *vulnificus*, *1 Vibrio* vent) (S1 Table). Strains *Escherichia coli* CECT4076 and *Salmonella enterica* Typhi CECT409 were also included in the analysis (S1 Table).

Results showed that all *V*. *cholerae* strains were efficiently identified by the qPCR (Cq-values ranging 22–23 at 10^5^ GU/rx) while all other *Vibrio* species showed negative Cq-values (Cq-values over 40 were considered negative). Electrophoresis analysis on 2% agarose gels stained with ethidium bromide confirmed the absence of non-specific products or primer dimers.

To assess if the method is affected by interference from non-target DNA, a condition commonly found in direct PCR analysis of complex DNA samples, proportional concentrations of *V*. *cholerae* genomic DNA (from 10^1^ to 10^4^ GU/μl) were tested in the presence of purified DNA from *V*. *mimicus* UM 6812 that was added to the qPCR reaction mixture at a concentration of 0.5 ng/rx (corresponding to 8x10^4^ GU/rx). Results showed that the qPCR protocol specifically detected target DNA also in the presence of *V*. *mimicus*. *V*. *cholerae* DNA was detected at similar Cq-values to those observed when performing the reaction in the absence of non-target DNA (Table 1). Moreover Cq-values were positively correlated with *V*. *cholerae* proportions (R^2^ = 0.98), indicating that non-target DNA did not interfere with the *gbpA* gene amplification. These results appear to be of particular relevance if we consider that *V*. *mimicus* is phylogenetically the most closely related species to *V*. *cholerae*, having been previously considered as a biotype of this species [38–40]. The protocol we developed not only discriminates between the two closely related species but is also not affected by interference of closely related DNA, suggesting its potential valuable use in complex samples (e.g. environmental samples) where DNA of the target organism would likely be present amongst a large number of different microbial cells.

**Table 1:** qPCR detection of *V*. *cholerae* ATCC 39315 cells in mixed cultures containing *V*. *mimicus* UM 6812.

*V*. *cholerae* concentration	Cq
10^4^ GU/μl	26.15±0.35
10^4^ GU /μl+*V*. *mimicus* DNA	26.15±0.49
10^3^ GU /μl	29.35±0.21
10^3^ GU /μl+*V*. *mimicus* DNA	29.40±0.42
10^2^ GU /μl	32.95±1.06
10^2^ GU /μl+*V*. *mimicus* DNA	32.90±0.14
10^1^ GU /μl	36.9±0.71
10^1^ GU /μl+*V*. *mimicus* DNA	36.75±0.35

qPCR detection of *V*. *cholerae* ATCC 39315 cells in mixed cultures containing 0.5 ng/rx (corresponding to 8x10^4^ GU/rx) of *V*. *mimicus* UM 6812. Quantification cycle (Cq) is expressed as mean ± standard deviation calculated from two spiking experiments each quantified in triplicate on the same run (n = 6)

### Standard curves and limit of detection with pure cultures

**Fig 1:**
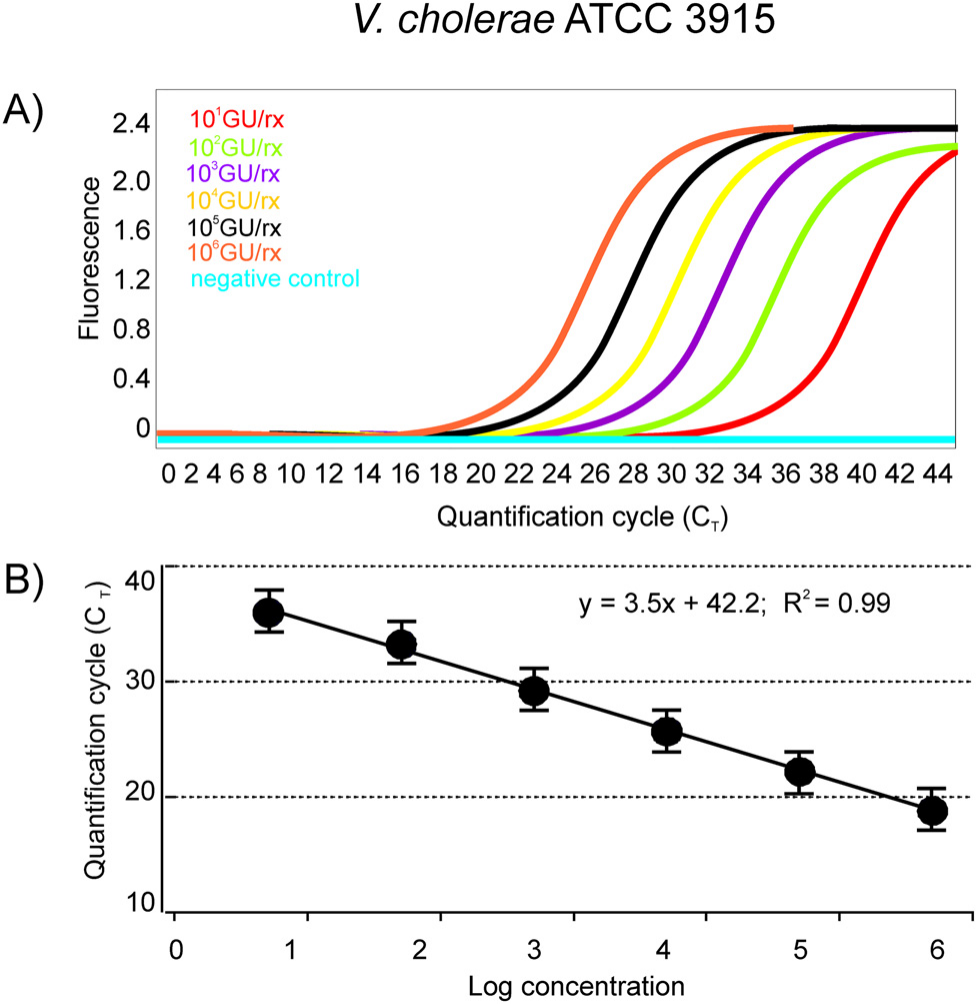
Sensitivity of the qPCR assay for detection of *V*. *cholerae* O1 El Tor N16961. DNA was amplified with the gbpA TaqMan primers in the presence of the gbpA fluorogenic probe. (A) Amplification plot of *V*. *cholerae* ATCC 39315 sample dilutions containing 10^6^, 10^5^, 10^4^, 10^3^, 10^2^, 10^1^ genome copies per reaction (GU/rx). (B) Plot of mean Cq-values from three replicates tested against the *V*. *cholerae* DNA inputs. Error bars indicate the standard deviations of the means.

Standard curves for the qPCR protocol were generated by preparing serial ten-fold dilutions of known concentrations (10^6^–10^0^ GU/rx) of *V*. *cholerae* ATCC 39315 genomic standard DNA (Fig 1). The amplification efficiencies of the qPCR assays were in the range of 82–116% and were able to detect the *gbpA* gene down to levels of 50 gene copies (50 genome equivalents considering that a single copy of the *gbpA* gene is present in the *V*. *cholerae* genome) (Fig 1).

Linearity was optimal (*R* ^2^ > 0.99) over a 6-log-unit dynamic range with a coefficient (calculated on genome copy values) of intra-assay variation of < 5% and the inter-assay variation of < 15%.

### Performance of *V*. *cholerae* qPCR in environmental and stool samples

The sensitivity of the method and amplification efficiency were evaluated on DNA extracted from different environmental matrices (drinking water, sea water, freshwater sediment, marine sediment, bivalve flesh) and stool samples spiked with known concentrations of *V*. *cholerae* ATCC 39315 cells (10^6^–10^3^ cells/l for drinking and sea surface water; 10^6^–10^1^ cells/g for all other matrices). Quantitative amplification parameters of the performed assays are reported in Table 2. Linearity for all the assays was satisfactory (*R* ^2^ > 0.98) over a 5-log-unit dynamic range and the overall PCR amplification efficiencies ranged from 0.89 to 1.28. Detection limits corresponding to the smallest amount of template DNA resulting in positive amplification were of 10^2^ GU/l for drinking water and sea surface water, 10^1^ GU/g for freshwater and marine sediments and 10^2^ GU/g for mussel flesh and stool samples (Table 2). Generally, it was found that the qPCR performance (e.g. efficiency and sensitivity) in DNA extracted from seawater and drinking water samples was similar to that obtained with DNA extracted from a pure bacterial culture, thus showing no inhibition of the amplification. In contrast, the performance of the qPCR assay was slightly lower, but overall good, in sediment samples where PCR amplification can be severely hampered by the presence of inhibitory substances, which are co-extracted with nucleic acids, such as humic acids, other organic polymers and clay particles [33, 41]. Aquatic sediments are putative environmental reservoirs for *V*. *cholerae* as it was recently shown that *Vibrio* bacteria, including the species *V*. *cholerae*, can be found at concentrations up to an order of magnitude higher in sediment than in seawater [42]. Although, to date, the role played by aquatic sediments in the persistence and spreading of toxigenic *V*. *cholerae* is not entirely clear, research must rely on methods able to provide rapid and quantitative estimates of the bacteria in this problem area. The qPCR protocol developed in this study shows a good performance and can be employed for detection and quantification of *V*. *cholerae* cells in aquatic sediment samples.

**Table 2:** Amplification parameters for the qPCR assay applied to environmental and stool samples.

Sample	Efficiency	Slope	Y intercept	R^2^	Linear range	Detection limit
**Drinking water**	100±11	-2.91±0.14	41.1±0.5	>0,98	10^6^–10^1^ GU/L	10^2^ GU/L
**Sea water**	100±15	-3.02±0.16	40.9±0.6	>0,98	10^6^–10^1^ GU/L	10^2^ GU/L
**Freshwater sediment**	113±5	-3.06±0.12	38.7±0.5	>0,99	10^6^–10^1^ GU/gr	10^1^ GU/g
**Marine sediment**	112±12	-3.07±0.18	38.9±0.4	>0,99	10^6^–10^1^ GU/gr	10^1^ GU/g
**Mussels flesh**	105±9	-3.21±0.14	46.9±0.4	>0,99	10^6^–10^1^ GU/gr	10^2^ GU/g
**Stool**	83±13	-3.82±0.12	46.9±0.7	>0,99	10^6^–10^1^ GU/gr	10^2^ GU/g

The values of Efficiency, Slope and Intercept are expressed as mean ± standard deviation calculated from two spiking experiments with *V*. *cholerae* ATCC 39315 cells, each quantified in triplicate on the same run (n = 6)

In mussel flesh the sensitivity of the qPCR showed a detection limit (10^2^ GU/g), around one log—unit lower than values obtained for the sediment but similar to limits reported in other studies obtained with a qPCR assay for endpoint detection [16]. Again this can be dependent upon the presence of PCR inhibitors in these samples. The qPCR assay may therefore be considered a useful tool for rapid and specific detection of *V*. *cholerae* in harvested and post-harvested bivalve molluscs.

The same sensitivity value of 10^2^ GU/g was also observed when performing the analysis on stool samples. Because the abundance of *V*. *cholerae* cells in cholera-affected humans is normally ranging from 10^7^ to 10^8^ CFU/g and up to 10^11^ to 10^13^ CFU/g or more in case of severe cholera [3], it can be inferred that the qPCR developed in this study should be also sufficiently sensitive to directly detect *V*. *cholerae* from diarrheal stool specimens. Furthermore, considering that the *V*. *cholerae* load in stools from convalescent and long-term carriers is reportedly in the range of 10^2^ to 10^3^ CFU/g [43, 44] the method is also valuable for the bacterium detection in these samples.

It has finally to be considered that, apart from CPR samples (see next paragraph), all experiments in this study are based on artificially spiked samples. This means that for matrices like animal tissues, stool or environmental samples, the detection limit might be different to artificially spiked samples (e.g. depending upon the different extraction efficiency, presence of non-target DNA, inhibitors, etc). For such problematic samples the use of the LightMix Modular PhHV spiked Extraction Control specifically designed for the LightCycler may be considered as a spiked internal control in a further development of the current qPCR protocol.

### Performance of *V*. *cholerae* qPCR in formalin-fixed and degraded samples

Fixation with formalin, a 37–40% (w/v) of formaldehyde gas in water, has traditionally been used for the fixation of biological and environmental samples for subsequent microscopic analyses and other studies.

However, nucleic acids isolated from formalin-fixed samples are most often degraded and contain small DNA fragments, generally less than 300 bp [45, 46]. Such small fragments make DNA often inaccessible to the PCR reaction [47, 48]. The fact that the developed primers amplify a short fragment of 206 bp makes them suitable also for the analysis of formalin-fixed samples.

The performance of the method was evaluated by applying the qPCR on genomic DNA extracted for a dilution series of 4% formalin-fixed *V*. *cholerae* ATCC 39315 spiked suspensions (10^6^–10^1^ GU/rx) using the protocol we previously described for molecular analysis of historical CPR samples [25]. This protocol included specific steps such as incubation at an elevated temperature after proteinase K digestion that partially removes formalin crosslinking (which is also a result of formalin-fixation, leading to decreases in the accessibility of extracted DNA for enzymes such as DNA polymerase during PCR) thus allowing genomic DNA to be efficiently purified for subsequent molecular analyses [25]. As shown in Table 3, the qPCR was able to detect and quantify target DNA down to a level of 10^1^ GU/rx (50 gene copies per reaction). The linearity of the performed assays was good (*R* ^2^ > 0.99) over a 6-log-unit dynamic range and the PCR amplification efficiencies ranged from 0.94 to 1.04.

**Table 3:** Amplification parameters for the qPCR assay applied to artificial formalin-fixed samples.

Sample	Efficiency	Slope	Y intercept	R^2^	Linear range	Detection limit
**ASW**	104±6	-3.22±0.12	41.4±0.4	>0,99	10^6^–10^1^ GU/rx	10^1^ GU/rx
**4% formalin 1 week**	94±9	-3.46±0.12	41.9±0.4	>0,99	10^6^–10^1^ GU/rx	10^1^ GU/rx
**4% formalin 2 week**	96±7	-3.43±0.08	42.3±0.8	>0,99	10^6^–10^1^ GU/rx	10^1^ GU/rx
**4% formalin 4 week**	94±6	-3.47±0.13	43.4±0.3	>0,99	10^6^–10^1^ GU/rx	10^2^ GU/rx
**4% formalin 8 week**	103±4	-3.25±0.09	43.3±0.3	>0,99	10^6^–10^1^ GU/rx	10^2^ GU/rx

The values of Efficiency, Slope and Intercept are expressed as mean ± standard deviation calculated from two spiking experiments with *V*. *cholerae* ATCC 39315 cells, each quantified in triplicate on the same run (n = 6)

**Fig 2:**
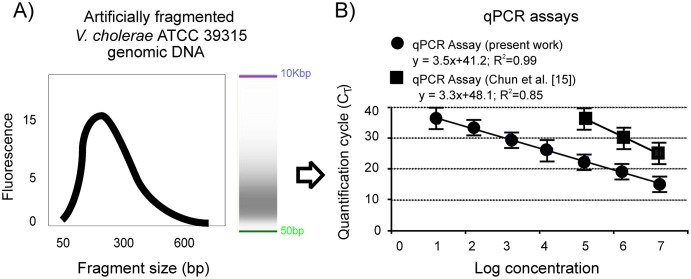
Performance of the qPCR assay for detection of *V*. *cholerae* in artificially degraded DNA samples. (A) Electropherogram plot obtained by Agilent Bioanalyzer analysis of artificially fragmented genomic DNA of *V*. *cholerae* ATCC 39315. (B) Plot of mean Cq-values from three replicates tested against the *V*. *cholerae* artificially fragmented DNA inputs. Error bars indicate the standard deviations of the means.

The qPCR was also performed on serial dilutions (10^6^–10^1^ GU/rx) of artificially fragmented *V*. *cholerae* ATCC 39315 genomic DNA samples composed of a homogenous pool of fragments having an average size of 276 bp (Fig 2A). This level of fragmentation was specifically selected in order to mimic DNA degraded conditions commonly encountered in DNA retrieved from historical formalin-fixed samples [45, 46] including CPR samples [25]. As shown in Fig 2B, qPCR performance on fragmented DNA was very good in terms of efficiency (0.91), linearity (*R* ^2^ > 0.99) and limit of detection (10^1^ GU/rx) and comparable to the performance of the method with pure-culture DNA. In addition, the performance of the method with fragmented samples was much better than performance by one of the most commonly employed qPCR protocol applied on the same samples [15] (Fig 2B).

### A case study: *V*. *cholerae* detection in Continuous Plankton Recorder (CPR) samples

The performance of the method was ultimately evaluated on historical formalin-fixed samples collected by the CPR survey during the last 60 years in several worldwide locations. The CPR Survey is one of the longest running marine biological monitoring programs in the world and provides a long-term archive of formalin-preserved plankton samples (http://www.sahfos.ac.uk). Because the plankton is one of the largest reservoirs of vibrios in nature, the molecular analysis of CPR samples has recently been proposed as a breakthrough approach to study the long-term ecology (over large temporal scales) and macro-ecology (over large spatial scales) of vibrios in the aquatic environment [49].

To test for the presence of PCR inhibitors the LightMix Modular PhHV spiked Extraction Control (Roche Diagnostics, Milan, Italy) was applied on DNA recovered from representative CPR samples (all samples scored negative for the qPCR test) collected at different times and geographic locations: 11IN2-2 (Irish Sea, 1971), 157SB-38 (Iberian Coast, 1971), 4CT4 (South Africa, 2011), 8CT30 (South Africa, 2011). A 85 bp long fragment from the Phocine herpesvirus (PhHV) sequence target was amplified with specific primers and detected with a LC670 labeled hydrolysis probe. Results from the test showed that no inhibitors were present in the tested DNA.

**Fig 3:**
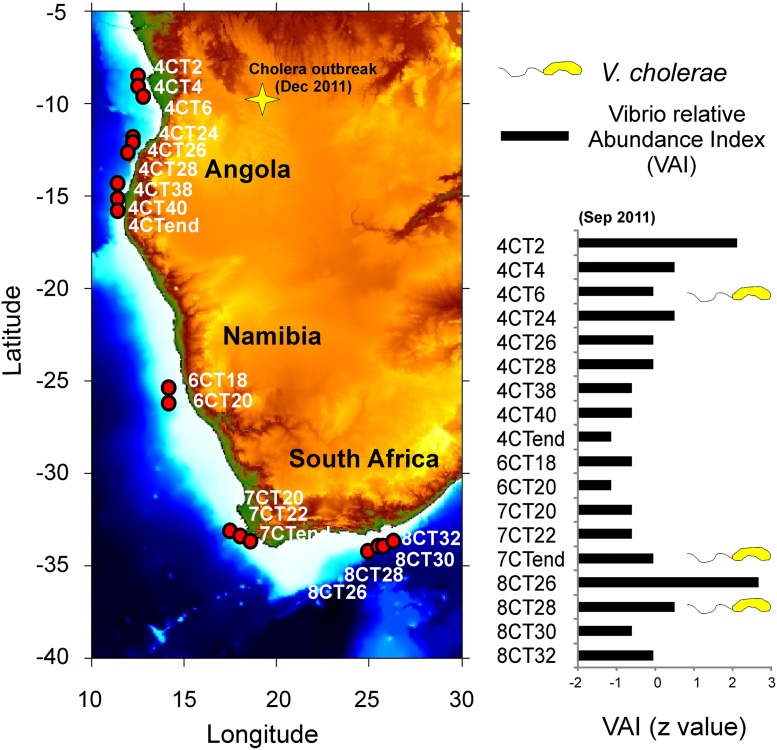
*V*. *cholerae* detection in CPR samples from endemic Cholera regions (South West Africa). Relative abundance of *Vibrio* spp. and *Vibrio cholerae* in CPR samples collected in cholera endemic areas of the Benguela Current Large Marine Ecosystem region (BCLME, South West Africa) as a proof of concept for the possible use of the CPR technology and the developed qPCR assay in cholera studies (see main text for details).

qPCR was firstly applied on two samples (413R and 485R) collected in the North Sea off the river Rhine estuary (51.9–52.41N; 3.3–4.01E) in 1998 and 2004, respectively. These samples were analyzed in a previous study by 16SrDNA amplicon pyrosequencing that revealed the presence of read sequences showing >95% identity to *V*. *cholerae* [25]. According to these results both 413R and 485R samples tested positive for qPCR. The method was then applied on 5 additional CPR samples that were collected by the Survey between 1961 and 1971 at different coastal locations of the North Sea and North Atlantic Ocean (S2 Table). Sample 157SB from the Bay of Biscay (47.5–48.5N; 4.5–5.5 W) in 1971 and sample 228A collected in the vicinity of the Shetland Islands (58–59N; 1–2 W) in 1966 scored positive to the test (S2 Table). Finally, the qPCR was run on 18 CPR samples collected in 2011 along the coasts of Angola, Namibia and South Africa, which represent endemic areas for cholera (Fig 3). Interestingly, sample 8CT28 (Cq = 32) collected in coastal water off the city of Port Elizabeth (South Africa), sample 7CTend (Cq = 33) collected off the city of Cape Town (South Africa) and sample 4CT6 (Cq = 33) collected off the city of Luanda (Angola) were positive to the test. All positive results (samples 413R-, 485R-, 228A-8, 157SB-2, 8CT28, 7CTend, 4CT6) were further confirmed through purification and sequencing of the amplified fragments showing that amplicon sequences fully matched (100% coverage and 100% identity) the *gbpA* gene sequence of *Vibrio cholerae* reference strain N16961 (GenBank accession number: EU072441.1).

Collectively, these results demonstrate the success of the developed assay in detecting *V*. *cholerae* DNA in historical formalin-fixed samples, the earliest of which dates back to August 1966. This finding may have important implications for the study of long-term ecology of *V*. *cholerae* in the aquatic environment including investigations aiming to shed light on the effect of climate and environmental changes on the worldwide spreading of this bacterium and its associated diseases [49].

Broadly, the protocol may also be useful for the preliminary screening of *V*. *cholerae* O1, O139, Non-O1, and Non-O139 in formalin-fixed samples of particular ecological, evolutionary and/or epidemiological significance prior to being further analysed by the latest genotyping techniques such as whole genome targeted capture-enrichment methods coupled to next generation sequencing (NGS) approaches [50]. In this context, the detection of *V*. *cholerae* in three out of eighteen CPR samples collected in cholera endemic areas (e.g. sample 8CT28, 7CTend and 4CT 6), is of particular significance and represents a proof of concept for the possible use of the CPR technology in cholera studies (Fig 3).

Although “absolute quantification” of bacterial cells could not be attempted on CPR samples (due to the loss/damage of DNA that could not be taken into account), “relative quantification” such as the use of the VAI index may be applied (Fig 3). Such an index, which was initially developed for the relative quantification of total vibrios in CPR samples [25], could be extended to measure and compare *V*. *cholerae* relative abundances in different geographic areas and time periods. For instance, CPR sampling and analysis of plankton and *V*. *cholerae* might be employed to study, for the first time, the macro-ecology of cholera, i.e. the relationships between the bacterium and its environment at a geographically extensive scale. Our understanding of such relationships has been constrained in the past mainly because sampling of *V*. *cholerae* in the environment was only obtained at single point sites and times, which do not capture the ecological background of the disease.

The use of the CPR in tracking cholera outbreaks and epidemics is also of great potential interest if we consider that one CPR sample alone (representing a tow over 10 nautical miles) is equivalent to multiple point samples typically employed in environmental cholera studies, thus providing orders of magnitude improvement in sampling coverage. Against this background, it is worth mentioning that more than 100 cases of cholera were registered during the first fortnight of December 2011 (2 months after the collection of CPR samples analyzed in this study) in the district of Lucapa (northern Angola) located 800 km away from Luanda (Fig 3). Genotyping of *V*. *cholerae* DNA recovered from this sample is currently in progress in our laboratory, although, at this stage, no direct or indirect connection can obviously be inferred.

Cholera research by the Continuous Plankton Recorder Survey is a collaborative development between the University of Genoa and the Sir Alister Hardy Foundation for Ocean Science (SAHFOS) and is expected to open up new research avenues addressing some important questions such as the role of human versus environmental factors in the origin, transmission and spreading of the cholera disease.

## Conclusions

Overall the qPCR protocol developed in this work provides a new tool for the robust and sensitive detection and quantification of *V*. *cholerae* in problematic matrices such as environmental and stool samples. This has potential application for studies investigating the ecology and epidemiology of *V*. *cholerae* and in the field of public health. In addition, the method performs very well on formalin-fixed biological samples and is of great value for a wide range of applications including genetic, evolutionary, biogeographic, ecological and epidemiological retrospective studies. As a case study, the detection of *V*. *cholerae* in CPR samples collected in cholera endemic areas such as the Benguela Current Large Marine Ecosystem (BCLME) is of particular significance and represents a proof of concept for the possible use of the CPR technology and the developed qPCR assay in cholera studies.

## Supporting Information

S1 Table Species and strains tested. (PDF)

S2 Table qPCR detection of *Vibrio cholerae* in Continuous Plankton Recorder samples. (PDF)

## References

[1] WHO (2014) Cholera 2013. Weekly Epidemiological Record. 2014; 89 (31): 345–356 25136711

[2] Vezzulli L, Pruzzo C, Huq A, Colwell RR. Environmental reservoirs of *Vibrio cholerae* and their role in cholera. Env Microbiol Rep. 2010;2: 27–33. 10.1111/j.1758-2229.2009.00128.x 23765995

[3] Sack DA, Sack RB, Nair GB, Siddique AK. Cholera. Lancet. 2004;17: 223–233 10.1016/s0140-6736(03)15328-7 14738797

[4] Colwell RR. Global climate and infectious disease: the cholera paradigm. Science. 1996;274: 2025–2031. 8953025 10.1126/science.274.5295.2025

[5] Bopp CA, Ries AA, Well JG. Laboratory Methods for the Diagnosis of Epidemic Dysentery and Cholera. 1999; Centers for Disease Control and Prevention.

[6] Choopun N, Louis V, Huq A, Colwell RR. Simple procedure for rapid identification of *Vibrio cholerae* from the aquatic environment. Appl Environ Microbiol. 2002;68:995–998. 11823252 10.1128/AEM.68.2.995-998.2002 PMC126716

[7] Tamrakar AK, Goel AK, Kamboj DV, Singh L. Surveillance methodology for *V*. *cholerae* in environmental samples. Int J Environ Health Res. 2006;16(4): 305–312. 16854675 10.1080/09603120600734303

[8] Huq A, Rivera ING, Colwell RR. Epidemiological significance of viable but nonculturable microorganisms In: Colwell RR and Grimes DJ, editors. Nonculturable microorganisms in the environment. Washington D.C.: American Society for Microbiology 2000 pp. 301–323.

[9] Oliver JD. The viable but nonculturable state in bacteria. J Microbiol. 2005;43: 93–100. 15765062

[10] Schauer S, Sommer R, Farnleitner AH, Kirschner AK. Rapid and sensitive quantification of *Vibrio cholerae* and *Vibrio mimicus* cells in water samples by use of catalyzed reporter deposition fluorescence in situ hybridization combined with solid-phase cytometry. Appl Environ Microbiol. 2012;78(20): 7369–7375. 22885749 10.1128/AEM.02190-12 PMC3457089

[11] Kurakawa T, Kubota H, Tsuji H, Matsuda K, Asahara T, Takahashi T, et al. Development of a sensitive rRNA-targeted reverse transcription-quantitative polymerase chain reaction for detection of *Vibrio cholerae/mimicus*, *V*. *parahaemolyticus/alginolyticus* and *Campylobacter jejuni/coli* . Microbiol Immunol. 2012;56(1): 10–20. 10.1111/j.1348-0421.2011.00405.x 22146006

[12] Hossain MT, Kim EY, Kim YR, Kim DG, Kong IS. Development of a groEL gene-based species-specific multiplex polymerase chain reaction assay for simultaneous detection of *Vibrio cholerae*, *Vibrio parahaemolyticus* and *Vibrio vulnificus* . J Appl Microbiol. 2013;114(2): 448–56. 10.1111/jam.12056 23121500

[13] Neogi SB, Chowdhury N, Asakura M, Hinenoya A, Haldar S, Saidi SM, et al A highly sensitive and specific multiplex PCR assay for simultaneous detection of *Vibrio cholerae*, *Vibrio parahaemolyticus* and *Vibrio vulnificus* . Lett Appl Microbiol. 2010;51(3): 293–300. 10.1111/j.1472-765X.2010.02895.x 20666989

[14] Fakruddin MD, Sultana M, Ahmed MM, Chowdhury A, Choudhury N. Multiplex PCR (polymerase chain reaction) assay for detection of *E*. *coli* O157:H7, *Salmonella* sp., *Vibrio cholerae* and *Vibrio parahaemolyticus* in spiked shrimps (*Penaeus monodon*). Pak J Biol Sci. 2013;16(6): 267–274 24498789 10.3923/pjbs.2013.267.274

[15] Chun J, Huq A, Colwell RR. Analysis of 16S-23S rRNA Intergenic Spacer Regions of *Vibrio cholerae* and *Vibrio mimicus* . Appl Environ Microbiol. 1999;65(5): 2202 10224020 10.1128/aem.65.5.2202-2208.1999 PMC91317

[16] Lyon WJ. TaqMan PCR for detection of *Vibrio cholerae* O1, O139, non-O1 and non-O139 in pure cultures, raw oysters and synthetic seawater. Appl Environ Microbiol. 2001;67(10): 4685–4693. 11571173 10.1128/AEM.67.10.4685-4693.2001 PMC93220

[17] Kim-Fatt L, Abdah K, Chan YY. A thermostabilized magnetogenosensing assay for DNA sequence-specific detection and quantification of *Vibrio cholerae* . Biosens Bioelectron. 2013;47: 38–44. 10.1016/j.bios.2013.03.004 23545172

[18] Fykse EM, Nilsen T, Nielsen AD, Tryland I, Delacroix S, Blatny JM. Real-time PCR and NASBA for rapid and sensitive detection of *Vibrio cholerae* in ballast water. Mar Pollut Bull. 2012;64(2): 200–206. 10.1016/j.marpolbul.2011.12.007 22221710

[19] Malayil L, Turner JW, Mote BL, Howe K, Lipp EK. Evaluation of enrichment media for improved PCR-based detection of *V*. *cholerae* and *V*. *vulnificus* from estuarine water and plankton. J Appl Microbiol. 2011;110(6): 1470–1475. 10.1111/j.1365-2672.2011.04996.x 21395948

[20] Lam JT, Lui E, Chau S, Kueh CS, Yung YK, Yam WC. Evaluation of real-time PCR for quantitative detection of *Escherichia coli* in beach water. J Water Health. 2014;12(1): 51–56. 10.2166/wh.2013.038 24642432

[21] Wang D, Xu X, Deng X, Chen C, Li B, Tan H, et al Detection of *Vibrio cholerae* O1 and O139 in Environmental Water Samples by an Immunofluorescent-Aggregation Assay. Appl Environ Microbiol. 2010;76(16): 5520–5525. 10.1128/AEM.02559-09 20581193 PMC2918967

[22] Ripley SJ, Baker AC, Miller PI, Walne AW, Schroeder DC. Development and validation of a molecular technique for the analysis of archived formalinpreserved phytoplankton samples permits retrospective assessment of Emiliania huxleyi communities. J Microbiol Meth. 2008;73: 118–124. 10.1016/j.mimet.2008.02.001 18358549

[23] Coombs NJ, Gough AC, Primrose JN. Optimisation of DNA and RNA extraction from archival formalin-fixed tissue. Nucleic Acids Res. 1999;27: 16. 10.1093/nar/27.16.e12 PMC148555 10454649

[24] Lister AM, Fenberg PB, Glover AG, James GE, Johnson KG. Natural history collections as sources of long-term datasets. Trends Ecol Evol. 2011;26(4): 153–154. 10.1016/j.tree.2010.12.009 21255862

[25] Vezzulli L, Brettar I, Pezzati E, Reid PC, Colwell RR, Höfle MG, et al Long-term effects of ocean warming on the prokaryotic community: evidence from the vibrios. ISME J. 2012;6(1): 21–30. 10.1038/ismej.2011.89 21753799 PMC3246245

[26] Zampini M, Pruzzo C, Bondre VP, Tarsi R, Cosmo M, Bacciaglia A, et al *Vibrio cholerae* persistence in aquatic environments and colonization of intestinal cells: involvement of a common adhesion mechanism. FEMS Microbiol Lett. 2005;244: 267–273. 15766778 10.1016/j.femsle.2005.01.052

[27] Kirn TJ, Jude BA, Taylor RK. A colonization factor links *Vibrio cholerae* environmental survival and human infection. Nature. 2005;438: 863–866. 16341015 10.1038/nature04249

[28] Stauder M, Huq A, Pezzati E, Grim CJ, Ramoino P, Pane L, et al Role of GbpA protein, an important virulence-related colonization factor, for *Vibrio cholerae*’s survival in the aquatic environment. Env Microbiol Rep. 2012;4(4): 439–445. 23760830 10.1111/j.1758-2229.2012.00356.x

[29] Hall TA. BioEdit: a user-friendly biological sequence alignment editor and analysis program for Windows 95/98/NT. Nucl Acids Symp Ser. 1999;41:95–98.

[30] Koressaar T, Remm M. Enhancements and modifications of primer design program Primer3 Bioinformatics. 2007;23(10):1289–1291. 17379693 10.1093/bioinformatics/btm091

[31] Untergrasser A, Cutcutache I, Koressaar T, Ye J, Faircloth BC, Remm M, et al Primer3—new capabilities and interfaces. Nucleic Acids Res. 2012;40(15): e115 22730293 10.1093/nar/gks596 PMC3424584

[32] Altschul S, Gish W, Miller W, Myers E, Lipman D. Basic local alignment search tool. J Mol Biol. 1990;215: 403–410. 2231712 10.1016/S0022-2836(05)80360-2

[33] Vezzulli L, Pezzati E, Moreno M, Stauder M, Fabiano M, Pruzzo C. Molecular ecology of marine sediments: determination of Real-Time PCR efficiency for quantifying microbial cells. Chem Ecol. 2009;25: 285–292.

[34] Reid PC, Colebrook JM, Matthews JBL, Aiken J. The Continuous Plankton Recorder: concepts and history, from Plankton Indicator to undulating recorders. Prog Oceanogr. 2003;58: 117–173.

[35] Thompson JR, Randa MA, Marcelino LA, Tomita-Mitchell A, Lim E, Polz MF. Diversity and dynamics of a North Atlantic coastal *Vibrio* community. Appl Environ Microbiol. 2004;70: 4103–4110. 15240289 10.1128/AEM.70.7.4103-4110.2004 PMC444776

[36] Sogin ML, Morrison HG, Huber JA, Mark Welch D, Huse SM, Neal PR, et al Microbial diversity in the deep sea and the underexplored ‘rare biosphere’. Proc Natl Acad Sci USA. 2006;103: 12115–12120. 16880384 10.1073/pnas.0605127103 PMC1524930

[37] Benson DA, Cavanaugh M, Clark K, Karsch-Mizrachi I, Lipman DJ, et al GenBank. Nucleic Acids Res. 2013;41:D36–42 10.1093/nar/gks1195 23193287 PMC3531190

[38] Davis BR, Fanning GR, Madden JM, Steigerwalt AG, Bradford HB, Smith HL Jr, et al Characterization of biochemically atypical *Vibrio cholerae* strains and designation of a new pathogenic species, *Vibrio mimicus* . J Clin Microbiol. 1981;14: 631–639. 7037833 10.1128/jcm.14.6.631-639.1981 PMC274012

[39] Wang D, Wang H, Zhou Y, Zhang Q, Zhang F, Du P, et al Genome Sequencing Reveals Unique Mutations in Characteristic Metabolic Pathways and the Transfer of Virulence Genes between *V*. *mimicus* and *V*. *cholerae* . Plos ONE. 2011;6(6): e21299 10.1371/journal.pone.0021299 21731695 PMC3120857

[40] Colwell RR. Global microbial ecology of *Vibrio cholerae* In: Belkin S, Colwell RR, editors. Ocean and Health Pathogens in the Marine Environment. New York: Springer-Verlag; 2005 pp. 297–305.

[41] Kirk JL, Beaudette LA, Hart M, Moutoglis P, Klironomos JN, Lee H, et al Methods of studying soil microbial diversity. J Microbiol Meth. 2004;58: 169–188. 10.1016/j.mimet.2004.04.006 15234515

[42] Vezzulli L, Pezzati E, Moreno M, Fabiano M, Pane L, Pruzzo C, et al Benthic ecology of *Vibrio* spp. and pathogenic *Vibrio* species in a coastal Mediterranean environment (La Spezia Gulf, Italy). Microb Ecol. 2009;58: 808–818. 10.1007/s00248-009-9542-8 19543938

[43] Utsalo SJ, Eko FO, Umoh F, Asindi AA. Faecal excretion of Vibrio cholerae during convalescence of cholera patients in Calabar, Nigeria. Eur J Epidemiol. 1999; 15(4): 379–381. 10414380 10.1023/a:1007541317104

[44] Azurin JC, Kobari K, Barua D, Alvero M, Gomez CZ, Dizon JJ, et al A long-term carrier of cholera: Cholera Dolores. Bull World Health Organ. 1967;37(5): 745–749. 5300877 PMC2554929

[45] Paabo S. “Ancient DNA: extraction, characterization, molecular cloning, and enzymatic amplification. Proc Natl Acad Sci USA. 1989;86(6): 1939–1943. 2928314 10.1073/pnas.86.6.1939 PMC286820

[46] Dietrich D, Uhl B, Sailer V, Holmes EE, Jung M, Meller S, et al Improved PCR Performance Using Template DNA from Formalin-Fixed and Paraffin-Embedded Tissues by Overcoming PCR Inhibition. Plos One. 2013;8(10): e77771 10.1371/journal.pone.0077771 24155973 PMC3796491

[47] De Giorgi C, Finetti Sialer M, Lamberti F. Formalin-induced infidelity in PCR-amplified DNA fragments. Mol Cell Probes. 1994;8: 459–462. 7700266 10.1006/mcpr.1994.1065

[48] Ripley SJ, Baker AC, Miller PI, Walne AW, Schroeder DC. Development and validation of a molecular technique for the analysis of archived formalin-preserved phytoplankton samples permits retrospective assessment of *Emiliania huxleyi* communities. J Microbiol Meth. 2008; 73: 118–124. 10.1016/j.mimet.2008.02.001 18358549

[49] Vezzulli L, Colwell RR, Pruzzo C. Ocean warming and spread of pathogenic vibrios in the aquatic environment. Microb Ecol. 2013;65(4): 817–825. 10.1007/s00248-012-0163-2 23280498

[50] Devault AM, Golding GB, Waglechner N, Enk JM, Kuch M, Tien JH, et al Second-pandemic strain of *Vibrio cholerae* from the Philadelphia cholera outbreak of 1849. New Engl J Med.2014; 370(4): 334–340. 10.1056/NEJMoa1308663 24401020

