# Supplementary material for: gbpA as a Novel qPCR Target for the Species-Specific Detection of Vibrio cholerae O1, O139, Non-O1/Non-O139 in Environmental, Stool, and Historical Continuous Plankton Recorder Samples
Source: PLoS One. 2015 Apr 27;10(4):e0123983. doi: 10.1371/journal.pone.0123983 (PMC4411143; doi:10.1371/journal.pone.0123983)
Supplement: S1 Table — (PDF) [file pone.0123983.s001.pdf]

Table S1. Species and strains tested

Scientific name	strain	Source	qPCR result
V. cholerae O1 El Tor	RC27	Unknown	Positive
	DK 70, CT+, VPI-	Clinical	Positive
	RC144, INDRE 91/1	Unknown	Positive
	CIRS 101	Unknown	Positive
	ATCC 14034	Clinical	Positive
	ATCC 14035	Clinical	Positive
V. cholerae O1 Classical	ATCC 11623	Unknown	Positive
	O395	Clinical	Positive
	CD 81	Clinical	Positive
Other *V. cholerae* O1 strains	071, 628	Unknown	Positive
	817	Clinical	Positive
	582	Clinical	Positive
	5556	Clinical	Positive
	189	Clinical	Positive
	175	Clinical	Positive
V. cholerae O139	MO45, ATCC 51394	Clinical	Positive
	HH 329	Unknown	Positive
	CO-391 NT-638 (I)	Unknown	Positive
	CO-393 NT-642	Unknown	Positive
	MO10	Clinical	Positive
	Y206, M203-60	Environmental	Positive
	S424	Unknown	Positive
V. cholerae non-O1/non-O139	DK 13: O37	Clinical	Positive
	TMS 22	Environmental	Positive
	TMA 135	Unknown	Positive
	TMA 92	Unknown	Positive
	TMA21	Environmental	Positive
	NIH 62	Environmental	Positive
	NIH 63	Environmental	Positive
	NIH 64	Environmental	Positive
	NIH 65	Environmental	Positive
	NIH 66	Environmental	Positive
	NIH 67	Environmental	Positive
	NIH 68	Environmental	Positive
	NIH 69	Environmental	Positive
	NIH 70	Environmental	Positive
	NIH 71	Environmental	Positive
	NIH 72	Environmental	Positive
	NIH 73	Environmental	Positive
	NIH 74	Environmental	Positive
	NIH 75	Environmental	Positive
	NIH 76	Environmental	Positive
	NIH 78	Environmental	Positive
	NIH 79	Environmental	Positive
	NIH 85	Environmental	Positive
	NIH 86	Environmental	Positive
	09-143-1A	Environmental	Positive
	09-143-4A	Environmental	Positive
	09-143-5A	Environmental	Positive
	09-143-1B	Environmental	Positive
	09-143-2B	Environmental	Positive
	09-143-3B	Environmental	Positive
	09-143-4B	Environmental	Positive
	09-143-5B	Environmental	Positive
	09-143-1C	Environmental	Positive
	09-143-2C	Environmental	Positive

	09-143-3C	Environmental	Positive
	09-143-4C	Environmental	Positive
	09-143-5C	Environmental	Positive
	09-231-2	Environmental	Positive
	09-231-3	Environmental	Positive
	09-304-2A	Environmental	Positive
	09-304-1B	Environmental	Positive
	09-304-2B	Environmental	Positive
	09-304-3B	Environmental	Positive
	09-304-4B	Environmental	Positive
	09-304-5B	Environmental	Positive
	09-154-1	Environmental	Positive
	09-131-5	Environmental	Positive
	09-131-3	Environmental	Positive
	09-152-1	Clinical	Positive
	09-152-2	Clinical	Positive
Other *V. cholerae* strains	060, 80-31	Unknown	Positive
	Usa 8	Unknown	Positive
	3 amoz	Unknown	Positive
	8 amoz	Unknown	Positive
	11 amoz	Unknown	Positive
	12 amoz	Unknown	Positive
	16 amoz	Unknown	Positive
V. aestuarianus	01/32	Unknown	Negative
	Ifremer 12/016	Unknown	Negative
	Ifremer 01/308	Unknown	Negative
	Ifremer 02/041	Unknown	Negative
V. alginolyticus	ValGenoa1	Environmental	Negative
	ValGenoa2	Environmental	Negative
	ValGenoa3	Environmental	Negative
	ValGenoa4	Environmental	Negative
V. anguillarum	VanGenoa5	Environmental	Negative
V. coralliilyticus	P1 LMG 23696	Environmental	Negative
	TAV24	Environmental	Negative
V. harveyi	ORM4	Unknown	Negative
	7890	Unknown	Negative
	VH2	Unknown	Negative
V. metecus	VmGenoa1	Unknown	Negative
V. mimicus	CP 192	Unknown	Negative
	CP 197	Unknown	Negative
V. parahaemolyticus	PMA 1.5	Environmental	Negative
	PMA 3.5	Environmental	Negative
	PMA 37.5	Environmental	Negative
	PMA 19.5	Environmental	Negative
	PMA 22.5	Environmental	Negative
	PMA 27.5	Environmental	Negative
	PMA 3316	Environmental	Negative
	PMA 16.5	Environmental	Negative
	PMA 45.5	Environmental	Negative
	PMA 79	Environmental	Negative
	PMA 112	Environmental	Negative
	PMA 337	Environmental	Negative
	PMA 339	Environmental	Negative
	PMA 109.5	Environmental	Negative
	PMA 2.5	Environmental	Negative
	VPE1	Environmental	Negative

	VPE2	Environmental	Negative
	VPE7	Environmental	Negative
	VPE17	Environmental	Negative
	VPE279	Environmental	Negative
V. parilis	CP35	Unknown	Negative
V. splendidus	LGP32	Unknown	Negative
	Bivalife 147	Unknown	Negative
	Bivalife 152	Unknown	Negative
	Bivalife 153	Unknown	Negative
	Bivalife 156	Unknown	Negative
V. tapetis	GDE1	Unknown	Negative
	LP2	Unknown	Negative
V. vulnificus	33149	Unknown	Negative
	27567	Unknown	Negative
Vibrio vent	Ex25	Environmental	Negative
Escherichia coli	CECT 4076	Clinical	Negative
Salmonella enterica Typhi	CECT 409	Clinical	Negative
