# Supplementary material for: gbpA as a Novel qPCR Target for the Species-Specific Detection of Vibrio cholerae O1, O139, Non-O1/Non-O139 in Environmental, Stool, and Historical Continuous Plankton Recorder Samples
Source: PLoS One. 2015 Apr 27;10(4):e0123983. doi: 10.1371/journal.pone.0123983 (PMC4411143; doi:10.1371/journal.pone.0123983)
Supplement: S2 Table — (PDF) [file pone.0123983.s002.pdf]

Table S2. qPCR detection of *Vibrio cholerae* in Continuous Plankton Recorder samples

Sample Set	Sample_id	Latitude	Longitude	Year	Month	Geographic Area	qPCR result
A	413R-	52.135	3.62	1998	August	Rhine Estuary	***Positive***
	485R-	52.16	3.73	2004	August	Rhine Estuary	***Positive***
B	2EB-2	44.39	-63.48	1961	August	Nova Scotia	Negative
	11IN2-2	53.52	-3.58	1971	August	Irish Sea	Negative
	228A-8	58.11	-1.55	1966	August	Shetland Island	***Positive***
	157SB-2	48.36	-5.27	1971	August	Bay of Biscay	***Positive***
	157SB-38	43.13	-9.44	1971	August	Iberian Coast	Negative
C							
	4CT 2	-8.59	12.57	2011	September	Angola	Negative
	4CT 4	-9.17	12.49	2011	September	Angola	Negative
	4CT 6	-9.37	12.46	2011	September	Angola	***Positive***
	4CT 24	-12.33	12.10	2011	September	Angola	Negative
	4CT 26	-12.53	12.06	2011	September	Angola	Negative
	4CT 28	-13.13	12.02	2011	September	Angola	Negative
	4CT 38	-14.51	11.43	2011	September	Angola	Negative
	4CT 40	-15.10	11.38	2011	September	Angola	Negative
	4CTend	nd	nd	2011	September	Angola	Negative
	6CT 18	-25.50	14.22	2011	September	Namibia	Negative
	6CT 20	-26.10	14.28	2011	September	Namibia	Negative
	7CT 20	-33.28	17.55	2011	September	South Africa	Negative
	7CT 22	-33.43	18.11	2011	September	South Africa	Negative
	7CTend	-33.51	18.18	2011	September	South Africa	***Positive***
	8CT 26	-34.13	25.12	2011	September	South Africa	Negative
	8CT 28	-34.08	25.36	2011	September	South Africa	***Positive***
	8CT 30	-34.01	25.59	2011	September	South Africa	Negative
	8CT 32	-33.54	26.21	2011	September	South Africa	Negative
